# Post-Exposure Prophylaxis Prescribing Practices in a Lyme Disease-Endemic Area

**DOI:** 10.3390/idr18010019

**Published:** 2026-02-14

**Authors:** Eun Bin Lee, Anna Schotthoefer, Philip Whitfield

**Affiliations:** 1 Department of Pharmacy, Marshfield Clinic Region–Sanford Health, Marshfield, WI 54449, USA; whitfield.philip@marshfieldclinic.org; 2 Marshfield Clinic Research Institute, Marshfield, WI 54449, USA; schotthoefer.anna@marshfieldresearch.org

**Keywords:** Lyme disease, doxycycline, prophylaxis, tick bite, prescribing patterns, demographic

## Abstract

Background/Objectives: The 2020 Infectious Diseases Society of America (IDSA) guidelines recommend a single 200 mg dose of doxycycline within 72 h of tick removal after a high-risk bite for Lyme disease prophylaxis. However, limited data are available on prescribing practices related to this recommendation in highly endemic Lyme disease areas. Methods: We conducted a retrospective chart review on adult patients (aged ≥ 18 years) who received a single dose of oral doxycycline for Lyme disease prevention for the period 2022–2024 within a rural Wisconsin health system. Patient and provider prescribing characteristics were evaluated. Manual data abstraction was performed on a random sample of 155 prescribing events to assess adherence to IDSA guidelines. Results: A total of 2404 prophylaxis prescriptions were identified; 44% were prescribed to older adults between 65 and 79 years of age, 54% were prescribed to males, and 66% were prescribed to patients living in rural areas. Prescriptions peaked in spring and summer months, consistent with the known seasonal trends in tick activity. Prescribing was distributed relatively evenly across provider types, with the majority (77%) of cases occurring in outpatient and urgent care settings. Upon manual abstraction, doxycycline was indicated in 12% with the remainder either classified as possibly indicated or not indicated due to suboptimal documentation and nonadherence. Conclusions: Our study identified high rates of incomplete documentation and uncertainty in guideline concordance in a Lyme-endemic health system, highlighting the opportunities to support evidence-based prescribing and to improve documentation practices.

## 1. Introduction

Lyme disease, caused by the spirochete *Borrelia burgdorferi*, represents one of the most prevalent vector-borne illnesses in the United States [1]. While the estimated likelihood of contracting Lyme disease following a tick bite is low, at approximately 2.6 percent, an estimated 476,000 cases are diagnosed annually [2,3]. Approximately 10% to 20% of patients treated with antibiotics for the disease may experience residual symptoms, including cognitive impairments and neurological dysfunctions, affecting quality of life [4]. The disease also imposes a significant financial burden on patients and the healthcare system, with an estimated $345–$968 million spent each year on patient management, medical expenses, and productivity losses [5].

Lyme disease prevention currently centers on recommendations to use personal protection practices that reduce tick exposure and the use of post-exposure chemoprophylaxis with doxycycline. Per the latter, the 2020 Infectious Diseases Society of America (IDSA) guidelines recommend a single 200 mg dose of doxycycline to be administered within 72 h of a high-risk tick bite, defined as a bite from an *Ixodes* spp. tick, in a highly endemic region, and when the tick attachment was for 36 h or longer [6]. These criteria were established based on Lyme transmission biology and the evidence available from clinical trials. For instance, a randomized–controlled trial by Nadelman et al. demonstrated a statistically significant risk reduction in erythema migrans by 87 percent when doxycycline was administered within the 72 h of tick removal [7]. The 72 h cutoff corresponds to the early incubation period of *Borrelia burgdorferi* in the skin following transmission and when the administration of a single dose of doxycycline is most likely to prevent the proliferation and migration of the spirochetes away from the site of the tick bite [8,9]. Similarly, *Borrelia burgdorferi* transmission is a time-dependent process that rarely occurs before 36–48 h following tick attachment, supporting the recommendation to use chemoprophylaxis for bites involving ≥36 h of tick attachment [6]. Chemoprophylaxis is not recommended in other clinical scenarios as the benefits of chemoprophylaxis may not outweigh its risks [6].

The prudent use of chemoprophylaxis is recommended because the inappropriate prescribing of doxycycline for low-risk tick bites may lead to antimicrobial resistance, or alterations in the human microbiome, diminishing its clinical meaningfulness [10,11,12]. These consequences could be harmful given that doxycycline is used for a wide range of indications (e.g., acne vulgaris, *Chlamydia trachomatis* infection, prophylaxis of some sexually transmitted infections).

A recent study examining medication utilization for post-tick bite doxycycline prophylaxis in the United States from 2010 to 2020 revealed that patients aged 65 years and older accounted for 53% of doxycycline prescriptions, and nearly 55% of prescriptions were dispensed to women. Children were less likely to receive prophylaxis despite being at a higher risk for Lyme disease, and males also appeared less likely to receive doxycycline prophylaxis despite their predominance among reported cases. High dispensing rates in non-endemic states also were observed, further suggesting potential discrepancies between Lyme disease risk and prescribing behavior [13]. However, limited data exists on the real-world clinical application of the IDSA post-exposure chemoprophylaxis recommendation.

This study aimed to describe current prescribing practices of prophylactic doxycycline for Lyme disease and evaluate prescriber adherence to 2020 IDSA guideline recommendations in a highly endemic region. The findings will help identify opportunities for practice evaluation and quality improvement and promote evidence-based prescribing.

## 2. Materials and Methods

This retrospective observational study was conducted at the Marshfield Clinic Region of Sanford Health, a large health system serving rural communities in Wisconsin and the upper peninsula of Michigan, a highly Lyme-endemic region. This study was limited to patients aged 18 and above. We searched the electronic health record to obtain prescription orders for a single dose of oral doxycycline, 200 mg or less, between 1 January 2022 and 31 December 2024. To be included in this study, the prescription order needed to indicate that it was for Lyme disease post-tick bite exposure prophylaxis, or the orders co-occurred in time with clinical encounters associated with ICD-10 codes (e.g., Lyme disease codes, A69.2X, or W57.XXXA, S00.XXXX codes for insect bite [nonvenomous]) or internal billing codes for Lyme disease or erythema migrans 7 days prior or 3 days after the prescription. Orders were excluded if they had non-Lyme indications or additional doxycycline, either IV or oral, prescribed on the preceding or following day.

The following patient and provider characteristics were electronically collected: patient age, sex, race and ethnicity, state of residence, rural versus urban status of residence based on U.S. Census Bureau 2020 Census Urban Areas, year and month of a tick bite, patient encounter setting, and provider type and specialty [14].

Manual chart reviews were conducted on a random selection of 155 records to verify the indication for a single doxycycline dose and to assess compliance with IDSA guideline recommendations for Lyme disease prophylaxis [6]. We reviewed clinical notes for information documented about the tick species, duration of tick attachment (in hours), and the amount of time elapsed after tick removal (in hours). A prescription order was classified either as “indicated,” “possibly indicated,” or “not indicated.” A prescription was considered indicated if all guideline criteria were met and possibly indicated when key tick exposure details were missing due to patient recall limitations or incomplete documentation but were otherwise met, leading to our uncertainty in guideline adherence. A prescription was considered not indicated if a clear discrepancy with the guidelines existed, such as when prophylaxis was prescribed following a reported bite by the tick species *Dermacentor variabilis*, the American dog tick, which is colloquially known as the wood tick in Wisconsin, and which is not a competent vector for *B. burgdorferi* [15]. Research Electronic Data Capture (REDCap) version 15.4.0 (Vanderbilt University, Nashville, TN, USA) was utilized for manual chart abstraction and data storage.

Descriptive statistics were used to summarize the frequency of post-tick bite prophylaxis orders by month, year, patient demographics and residence, clinical setting, and IDSA guideline indication status of the prescription.

## 3. Results

### 3.1. Prescribing Practices

**Figure 1:**
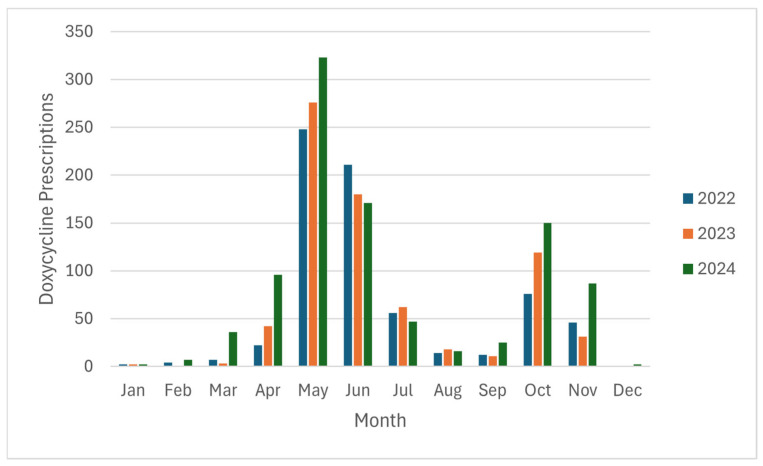
Monthly and yearly distribution of doxycycline prescriptions.

Between 1 January 2022 and 31 December 2024, a total of 2404 single-dose doxycycline prescriptions among 2251 patients meeting our inclusion criteria for receiving post-tick bite doxycycline prophylaxis were identified. The majority of patients (2125 of 2251; 94%) had a single order during the study time period. Of the 126 patients with more than one order, 104 (82%) had two orders, 18 (14%) had three orders, 3 (2%) had four orders, and 1 (0.8%) had five orders. Prescription counts varied by month, with peaks between May and June (35% and 23%), followed by October (14%) (Figure 1).

Of the 2404 prescriptions, 1051 (44%) were prescribed to older adults aged between 65 and 79. Prescriptions were lowest among young adults aged between 18 and 35, which accounted for 7% of prescriptions. More males (54% of prescriptions) than females received the prophylaxis for Lyme disease. Among the prescription recipients, 92% of the recipients were non-Hispanic White, 96% resided in Wisconsin, and 66% resided in rural areas (Table 1).

Prescribers were fairly evenly distributed across physicians (34%), nurse practitioners (35%), and physician assistants (29%). Patients were most frequently seen in the outpatient setting (44%), followed by the urgent care setting (32%; Table 2). Demographics and prescriber characteristic are further described in Table 1 and Table 2.

### 3.2. IDSA Guideline Adherence

Upon the review of 155 charts of patients prescribed doxycycline prophylaxis, a single-dose doxycycline prescription was indicated in 12% of cases, possibly indicated in 54%, and not indicated in 34%. We observed a suboptimal documentation of key guideline criteria. The time since tick removal was documented in 88% of cases; however, the duration of the tick attachment was only reported in 57% of cases, and tick identification was documented in 45% of cases. Of all prescription events reviewed, 98% involved a tick bite in a highly endemic area (e.g., in Wisconsin or Minnesota), 28% involved a tick that was attached for at least 36 h or engorged, 83% occurred within 72 h of tick removal, and 42% involved *Ixodes* spp. ticks (Table 3).

## 4. Discussion

This retrospective chart review of 2404 encounters with a single-dose doxycycline prescription analyzed the prescribing practices of post-exposure chemoprophylaxis for Lyme disease in a highly endemic area. Monthly prescribing patterns revealed a bimodal distribution, with peaks in May–June and October consistent with a combined level of activity of adult- and nymph-stage ticks [16].

Within the patient population examined in our study, older adults aged 65–79 most frequently received prophylaxis compared to other age groups. This finding is in line with a prior study by Marx et al., in which 53.8% of prescriptions were for older adults aged 65 or older across endemic and non-endemic states combined [13]. Lyme disease cases are commonly reported among older adults. This pattern may partly reflect a higher tendency to seek medical care, which may be related to their likelihood of having insurance coverage (e.g., Medicare) [17]. Our observation that more men than women received prophylaxis is also consistent with previously reported higher rates of Lyme disease among males (51–58% from 1992 to 2016, per Kugeler et al., based on Centers for Disease Control and Prevention [CDC] surveillance data) [17], but this is different from the report that more females than males received prophylaxis by Marx et al. [13]. Marx et al. [13] did not provide an explanation for why they may have observed a higher proportion of prophylaxis provided to women than men in their study. In our single health system study, a different direction of this difference was observed, and this heterogeneity in findings may warrant further investigation. The predominance of non-Hispanic White patients in our study reflects the local racial distribution, with 93.5% of the rural population identifying as White in 2022, as reported by the Wisconsin Office of Rural Health based on data from the U.S. Census Bureau [18]. Prophylaxis prescriptions were approximately evenly ordered by physicians, physician assistants, and nurse practitioners, most likely reflecting the distribution of these providers in ambulatory care practice. With licensure types relatively well represented across the ambulatory setting, the observed prescribing practices are unlikely biased by the provider composition.

Our results indicating a suboptimal adherence to current guidelines support the suggestion that prophylaxis may be overprescribed. The recommended criteria were not met in 88%, which was driven largely by incomplete documentation. Most commonly, there were gaps noted in tick identification and the duration of tick attachment. These criteria may be particularly challenging attributes for providers and patients to assess. For instance, a survey conducted to assess the proficiency of healthcare providers and trainees to correctly identify images of ticks by species and engorgement revealed that only about one-third of images were accurately identified by respondents. Engorged ticks, in particular, were difficult for providers to accurately identify [19]. Additionally, patients often do not bring in the ticks that may have been removed from bite sites for providers to identify, such that providers often must rely solely on patient-reported details without the ability to verify the information. Tick bites are also often painless as tick saliva counteracts bradykinin; a mediator responsible for pain [20]. Due to the absence of pain, patients frequently do not see when, or if, a tick is attached. Providers may err on the side of providing the protection rather than strictly adhering to the prophylaxis guidelines when patient-reported details are incomplete or unreliable. Doxycycline is inexpensive and has a relatively favorable safety profile. Consequently, providers may face a dilemma between a concern about withholding prophylaxis and the perceived low harm of single-dose doxycycline prophylaxis. Patient requests may be another determinant leading to the prescription of prophylaxis.

Several studies have investigated the risk of antimicrobial resistance associated with the post-exposure prophylaxis use of a single dose of doxycycline for sexually transmitted infections. In a study utilizing a *Galleria mellonella* (greater wax moth) larva model by Kenyon et al., a gradual increase in minimum inhibitory concentrations (MICs) was observed in a range of Gram-negative organisms with repeated doxycycline exposure over 7 days, most notably with *Klebsiella pneumoniae* [10]. Another study demonstrated a statistically significant increase in levels of tetracycline resistance genes from day 0 to month 6 of a single 200 mg dose doxycycline prophylaxis after condomless sex (median 42 doses) [11]. Although the selection pressure is likely lower with the intermittent use of a single dose of doxycycline for Lyme disease prophylaxis, cumulative exposure from repeated Lyme prophylaxis and use for other indications may increase the risk of resistance among non-target bacterial populations. Doxycycline use may also be associated with microbiome alterations. A study has shown that a 10-day course of doxycycline (150 mg/day) resulted in a lower fecal microbiota diversity and quantity of *Bifidobacterium animalis* [12]. The intermittent use of single-dose doxycycline did not show a significant change in gut commensal bacterial diversity by month 6 [11]. While the risk of dysbiosis may be less with intermittent use, as it allows a time for recovery, the overall evidence is limited, and caution should still be exercised. The inappropriate use of prophylaxis should be avoided, when possible, to mitigate the risk of resistance, microbiota disruption, and adverse effects.

Different approaches could be implemented to improve adherence to evidence-based practices. One potential option is to provide clinical decision support tools and internal guidance on tick identification and the degree of engorgement to support a guideline-based tick bite assessment. Providers should encourage patients to bring in ticks for identification and assessment for future visits. Automated documentation tools on electronic health record software that requires providers to enter key guideline criteria details could also be utilized to support documentation. Provider education on realistic Lyme transmission risks and situations with uncertain prophylaxis benefits (e.g., <36 h of tick attachment, antibiotic prescribed >72 h since tick removal) could be implemented to guide risk-based prescribing. These educational initiatives should be directed towards providers in the outpatient and urgent care settings, where the majority of prophylaxis decisions were made in our study. However, these findings may not be generalizable to all institutions and should be interpreted in the context of local practice patterns and institutional needs.

A key strength of our study is the assessment of the adherence to the criteria-based guidelines provided for Lyme disease prophylaxis [6], which has been rarely utilized in prior studies. Integrating comprehensive data resources, including diagnosis codes and the signatura (“sig”), to identify unique single-dose doxycycline cases rather than relying on insurance claims alone may facilitate a more accurate representation of treatment practices.

The limitations of this study include its possible limited generalizability to broader populations with varying healthcare access or risk profiles, as our study examined a single, predominately White non-Hispanic adult population. We did not assess prophylaxis in children even though children are at risk for Lyme disease, and a prior study suggested that children may be less likely to receive prophylaxis after a tick bite than adults [14]. Assumptions about the presumed locations of tick bites were necessary due to the limited available data. Tick bites were presumed to have occurred in Wisconsin unless clinical records indicated travel outside of Wisconsin at the time of the tick bite. Unless otherwise indicated in notes, we also assumed that tick bites were obtained near the homes when examining prophylaxis for patients in rural versus urban areas. It is possible that tick bites occurred at other locations for some patients. It is difficult to draw sweeping conclusions about guideline adherence as less than 7% of the total prescription encounters were manually abstracted. However, the primary objective of this study was to descriptively characterize prescribing practices rather than to conduct formal hypothesis testing. To mitigate potential selection bias, encounters included in the manual review were randomly selected. Additionally, our study relied on the interpretation of information as documented in medical charts, which is an indirect measure of provider knowledge and behavior. A future analysis that quantifies the impact of patient- and provider-specific factors on prophylaxis prescribing behavior through multivariable modeling may be needed to fully understand issues related to the suboptimal adherence to the guidelines.

## 5. Conclusions

Our study revealed that older adult patients and males were most likely to receive doxycycline post-tick bite Lyme disease prophylaxis in a highly endemic area, which is consistent with national epidemiologic patterns showing higher reported Lyme disease cases among males and older adults. However, we observed suboptimal documentation within the electronic health record, evidenced by high rates of doxycycline prophylaxis being “possibly indicated”. These findings highlight opportunities to support evidence-based prescribing with provider education and improved documentation practices within the electronic health record.

## Figures and Tables

**Table 1:** Summary of patient and seasonal characteristics associated with post-tick bite doxycycline prescription practices in the cohort.

Patient Characteristics (*N* = 2404)	*n*	%
Age, years		
18–35	165	6.9
36–50	312	13.0
51–64	637	26.5
65–79	1051	43.7
>79	239	9.9
Sex		
Male	1304	54.2
Female	1099	45.7
Unknown	1	<0.1
Race/ethnicity		
White, non-Hispanic	2217	92.2
Other, non-Hispanic	124	5.2
Hispanic	2	0.1
Unknown	61	2.5
State of residence		
Wisconsin	2312	96.2
Illinois	24	1.0
Minnesota	16	0.7
Michigan	11	0.5
Other	41	1.7
Setting of residence		
Rural	1586	66.0
Urban	532	22.1
Unknown	286	11.9
Year		
2022	698	29.0
2023	744	30.9
2024	962	40.0
Month		
January	6	0.3
February	11	0.5
March	46	1.9
April	160	6.7
May	847	35.2
June	562	23.4
July	165	6.9
August	48	2.0
September	48	2.0
October	345	14.4
November	164	6.8
December	2	0.1
Season ^1^		
Winter	19	0.79
Spring	1053	43.8
Summer	775	32.2
Fall	557	23.2

^1^ Winter: December, January, and February; Spring: March, April, and May; Summer: June, July, and August; and Fall: September, October, and November.

**Table 2:** Summary of provider characteristics across the study cohort.

Prescriber Characteristics (*N* = 2404)	*n*	%
Encounter setting		
Outpatient	1064	44.3
Urgent care	778	32.4
Emergency	137	5.7
Inpatient	6	0.3
Pharmacy ^1^	170	7.1
Telehealth	1	<0.1
Multiple	151	6.3
Unknown	97	4.0
Provider type		
Physicians	820	34.1
Physician assistant	697	29.0
Nurse practitioners	848	35.3
Multiple	22	0.9
Unknown	17	0.7

^1^ Prescription following telephone encounter for tick bite.

**Table 3:** Infectious Diseases Society of America (IDSA) guideline adherence for a random subset of prescriptions, determined by manual chart review.

Tick Bite Characteristics (*N* = 155)	*n*	%
Geographical region		
Highly endemic	152	98.1
Non-endemic	3 ^4^	1.9
Duration of tick attachment		
0–35 h ^1^	45	29.0
≥36 h ^2^	43	27.7
Not available	67	43.2
Time since tick removal		
≤24 h	89	57.4
>24 to ≤48 h	24	15.5
>48 to ≤72 h	16	10.3
>72 h	7	4.5
Not available	19	12.3
Tick identification		
*Ixodes scapularis* ^3^	65	41.9
Other	5	3.2
Not available	85	54.8
**Guideline Adherence (*N* = 155)**	***n***	**%**
Indication status		
Indicated	19	12.3
Possibly indicated	83	53.5
Not indicated	53	34.2

^1^ For 0–35 h or not engorged/partially engorged; ^2^ ≥36 h or engorged. ^3^ Deer, bear, and blacklegged tick. ^4^ One visiting from Colorado. Two had recent visits to Illinois.

## Data Availability

Access to patient data is restricted to protect patient confidentiality. De-identified data may be provided on a case-to-case basis upon requests made via research.data@marshfieldresearch.org.

## Abbreviations

The following abbreviations are used in this manuscript:

IDSA	Infectious Diseases Society of America
OspA	Outer Surface Protein A
REDCap	Research Electronic Data Capture
CDC	Centers for Disease Control and Prevention
MIC	Minimum Inhibitory Concentration
